# Recognising and mitigating LLM Pollution in online behavioural research

**DOI:** 10.1038/s41467-026-74621-9

**Published:** 2026-06-25

**Authors:** Raluca Rilla, Tobias Werner, Hiromu Yakura, Iyad Rahwan, Anne-Marie Nussberger

**Affiliations:** 1 Center for Humans and Machines, Max Planck Institute for Human Development, Berlin, Germany; 2 International Max Planck Research School on Learning, Institutions, and Future Evolution (LIFE), Berlin, Germany; 3 Department of Economics, University of Southampton, Southampton, UK; 4 Maynooth University, Department of Economics, Maynooth, Co. Kildare Ireland

**Keywords:** Interdisciplinary studies, Research data

## Abstract

Online behavioural research faces a growing methodological and epistemic threat as participants increasingly rely on large language models: *LLM Pollution*. Amid accumulating empirical evidence of contamination, we introduce a conceptual framework that distinguishes three variants — *Partial LLM Mediation*, *Full LLM Delegation*, and *LLM Spillover*. Their interaction distorts samples, biases inferences, and fuels an escalating methodological arms race. We outline mitigation strategies spanning researcher practices, platform accountability, and community adaptation.

## The emergence of large language model pollution

Fast, scalable access to diverse participants through online recruitment platforms such as Prolific or Amazon Mechanical Turk has enabled a revolution in behavioural research^1–3^. The promise of authentic human responses lies at the core of their appeal for researchers seeking to study authentic human cognition and behaviour. For instance, Prolific, one of the leading survey providers, advertises its services as “Easily examine human actions and behaviours with our 100% human, ID-checked participants” (see https://www.prolific.com/academic-researchers, last accessed March 10, 2026). Yet as large language models (LLMs) become increasingly capable and readily available, this promise is under growing threat, evidenced not only by participants reporting routine LLM-use in study tasks^4^, but also by empirical audits showing substantial rates of LLM-use^5,6^. We term this emerging phenomenon, in which LLMs are involved in online tasks intended to measure human responses, *LLM Pollution*. It represents a cause of several downstream effects on the integrity of online behavioural research that we discuss in this paper.

Recent experiences from our lab illustrate the immediacy of this threat. In a pilot study we conducted with participants recruited via Prolific, we tracked copying and pasting attempts on a page featuring an open-ended question and found that 45% of participants engaged in either or both actions, suggesting potential cases of *Partial LLM Mediation* or *Full LLM Delegation*. Many of these responses seemed LLM-generated, characterised by overly verbose summaries of instructions or distinctly non-human phrases such as “I don’t experience confusion in the same way humans do” (see Supplementary Note S1.1 for more details). These observations are also corroborated by recent work from Veselovsky and colleagues^6^, who found that even when participants were explicitly asked not to use LLMs in a crowdsource task, a substantial share (up to 24%) still did so. More broadly, recent studies combining automated classifiers, behavioural heuristics, and self-reports report varying prevalence estimates across platforms^5^, emphasising how difficult it is to reliably detect and prevent *LLM Pollution*.

While threats to data quality from automated bots are not entirely new, earlier concerns focused on technically less sophisticated and more easily detectable bots^7–12^. LLMs’ growing fluency and accessibility have, however, substantially amplified the problem by generating responses that are increasingly indistinguishable from those written by humans^13^. As such, *LLM Pollution* can arise through a range of participant behaviours^4^. Some may use LLMs innocently, for example, to translate instructions or to improve fluency. Others might rely on them more strategically, using them to automate responses or reduce effort on tedious tasks. In more extreme cases, participants may delegate the completion of entire studies to agentic LLMs, such as OpenAI’s GPT Agent, or open-source tools like Browser Use that can operate browsers, interpret screenshots, and navigate experiments without human oversight.

Most immediately, *LLM Pollution* is problematic because it obscures the origin of responses: researchers may unknowingly draw conclusions from outputs shaped or even entirely generated by LLMs rather than humans. Because LLM-responses often are overly fluent, less variable, and culturally biased, they can distort distributions, inflate effects, or mask individual differences^14,15^. Additionally, the growing difficulty of detecting LLM involvement creates diagnostic uncertainty that undermines confidence in exclusion decisions, complicates interpretation, and raises ethical concerns. This uncertainty, exacerbated by rapid advances in model capabilities, risks fuelling a perpetual arms race between researchers and increasingly sophisticated LLM-usage^16^. Beyond these immediate challenges, the ultimate epistemic risk is that behavioural research may no longer validly capture human cognition and behaviour at all^17^. This threatens not just the reliability but the very purpose of online studies in the behavioural sciences.

In the following, we identify three key variants through which *LLM Pollution* manifests: *Partial LLM Mediation*, *Full LLM Delegation*, and *LLM Spillover*. We then outline a set of mitigation strategies, ranging from practical design choices to broader institutional safeguards. Finally, we offer an outlook on the future of online experiments in an era where human and machine responses are increasingly entangled. By synthesizing early anecdotal evidence and conceptual distinctions emerging from our work, this Comment provides a forward-looking framework to guide cumulative empirical testing and collaborative, iterative refinement of mitigation strategies as *LLM Pollution* evolves.

## Three variants of *LLM Pollution*

**Fig. 1:**
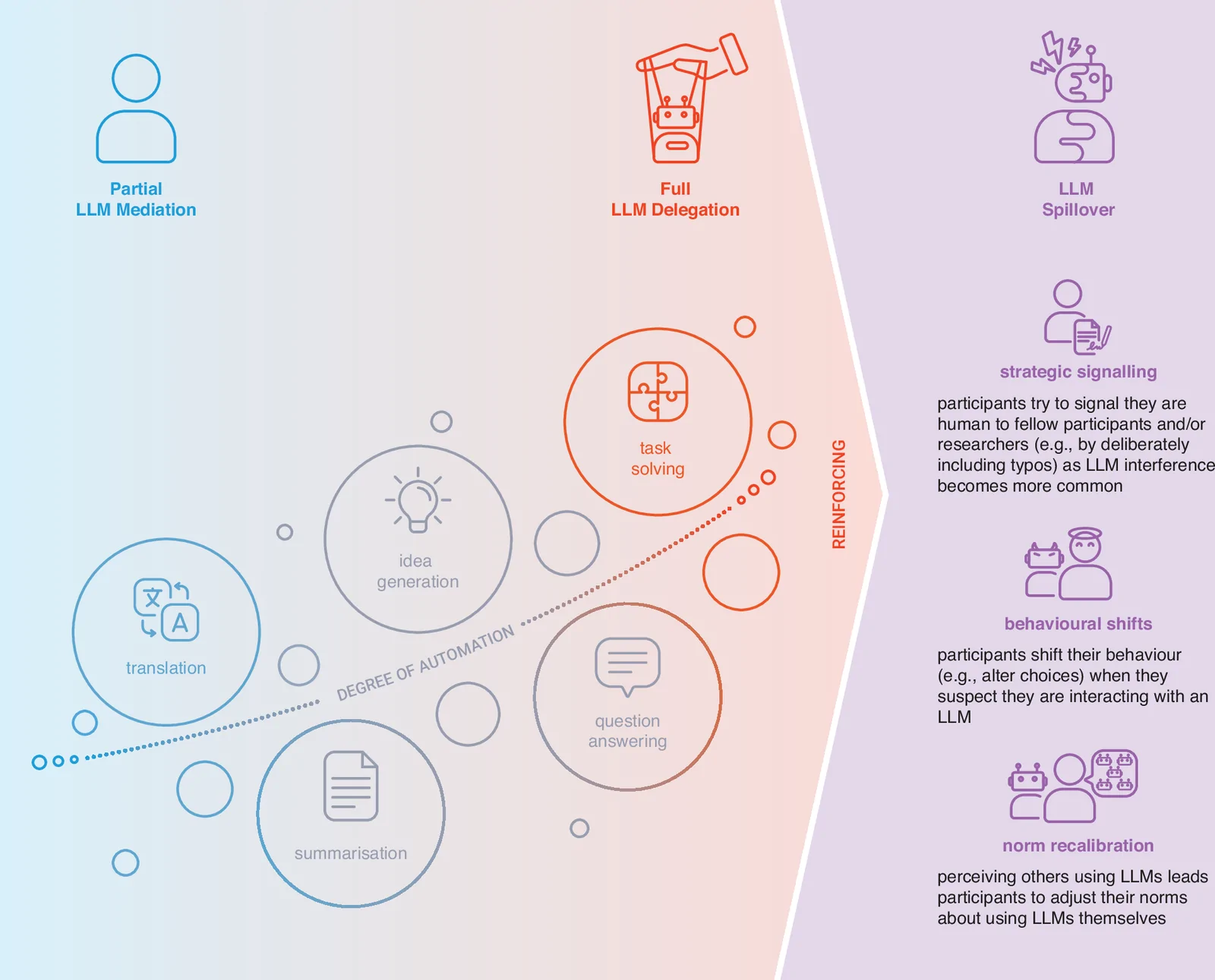
Three variants of *LLM Pollution*. *Partial LLM Mediation* refers to cases where participants use LLMs for translation, idea generation, or performance gains. *Full LLM Delegation* involves the use of agentic tools or plugins to automate participation entirely. *LLM Spillover* refers to normative or behavioural shifts prompted by participants' beliefs about LLM involvement, regardless of whether any is present. These variants can interact and reinforce one another, compounding their threat to research validity and inference. Illustration by H. Jahani.

We identify three distinct but interacting variants, visualised in Fig. 1, through which LLMs can pollute behavioural online experiments. Each poses a unique threat to the internal and external validity of experimental research.

First, *Partial LLM Mediation* arises when participants rely on LLMs to help refine or generate their responses. Such responses may then appear to be human-generated but are, in part, machine-shaped. For instance, participants may rely on LLMs to translate content, improve the fluency of their writing, reduce cognitive effort, provide strategic advice on how to complete the task efficiently, or to maximise payment. Participants may copy and paste study instructions into LLM interfaces such as ChatGPT, or use screen-reading agents like Gemini Live to receive guidance and mediate their participation in the experiment. In some open-ended responses, LLM involvement may be evident from overly fluent language or stylistic quirks typical of LLMs (see, for instance, work by ref. ^18,19^). Still, in many cases, *Partial LLM Mediation* remains difficult to detect from output alone, particularly when assistance is used only sparsely or intermittently. *Partial LLM Mediation* can compromise inference in several ways: because LLM-generated responses tend to exhibit lower variance and reflect dominant patterns in training data, their inclusion may artificially inflate central tendencies and obscure the true distribution of human responses^4^. These dominant patterns often encode systematic biases, such as the overrepresentation of Western, Educated, Industrialised, Rich, and Democratic linguistic and cultural norms^15,20^, which in turn are posed to produce homogenised outputs that mask meaningful variation, even within diverse participant samples. The decline of this naturally occurring diversity compromises the ecological and external validity of research findings, as responses reflect the linguistic regularities of training data rather than those of actual human populations. Researchers may believe they are analysing purely human-generated data, when in fact they are observing a blend of human and AI responses. This leads to incorrect inferences when the goal is to understand human cognition or behaviour. At the same time, *Partial LLM Mediation* has the potential to decrease internal validity by changing the very cognitive and linguistic processes that experimental manipulations aim to measure. Attempts to contrast human behaviour with machine behaviour may therefore rely on data that no longer represents the former in a clear manner.

The second variant, *Full LLM Delegation*, involves participants outsourcing the entire study interaction to LLM-based tools or agents. Though described separately for clarity, *Partial LLM Mediation* and *Full LLM Delegation* lie on a continuum of increasing automation, with gradual shifts and no clear cutoffs between levels of LLM involvement. Systems deployed in *Full LLM Delegation* typically combine a language model with a control interface that enables goal-directed interaction with web environments. In practice, browser-integrated LLM agents (e.g., OpenAI’s ChatGPT Agent, open-source solutions like Browser-Use, Skyvern, or Nanobrowser) can autonomously read study content, interpret instructions, click through consent forms, complete questionnaires, and generate both structured and open-ended responses—all with minimal or no human intervention once launched. This form of delegation is made even easier by new, dedicated AI browsers like Perplexity’s Comet and ChatGPT Atlas, which build agentic functionality directly into the browser. Moreover, these agentic LLMs are increasingly capable of identifying instructions that are designed to mislead or manipulate their behaviour (see Supplementary Fig. S1). They are built to operate autonomously but are also designed to permit temporary human handover when they encounter barriers, such as CAPTCHA or other bot-detection systems. Furthermore, participants often have the option to instruct LLMs to adopt specific standpoints, personas, or tones that make their responses appear more credible both in terms of content and formatting at the surface level^21^, further obscuring their underlying homogeneity^4^. *Full LLM Delegation* amplifies several risks observed in *Partial LLM Mediation*, as it more fully caters to participants’ motivations for efficiency and convenience, while distancing the resulting responses even further from genuine human behaviour. Hence, it first and foremost deepens the epistemic threat: researchers can no longer assume responses reflect human cognition, even probabilistically. Second, *Full LLM Delegation* may undermine treatment orthogonality, as LLMs may systematically respond differently across experimental conditions—especially in novel tasks—based on learned heuristics from training data^22,23^. Third, *Full LLM Delegation* enables scale: participants (or third parties) can deploy LLM agents across multiple studies simultaneously, transforming data collection platforms into sites of automated labour arbitrage. Affirming the validity of these concerns, recent modelling work has documented that synthetic respondents can now be constructed with relative ease in such a manner that they not only generate demographically plausible, psychometrically coherent, and behaviourally consistent responses at scale, but can also successfully bypass current detection methods and strategically distort aggregate response patterns^24^. Importantly, even in the absence of coordinated or strategic misuse of *Full LLM Delegation*, its mere possibility of scalable delegation to LLMs may reshape participant expectations and behaviour in ways that expand beyond direct model involvement.

We define the *LLM Pollution* variant that describes second-order effects of LLMs on human participants as *LLM Spillover*. Unlike *Partial LLM Mediation* or *Full LLM Delegation*, which focus on cases where LLMs are directly involved, *LLM Spillover* acknowledges that people’s mere expectations, prior experiences, and ambient awareness of LLMs may also shape their behaviour, even in the absence of direct model interactions. As such, *LLM Spillover* is likely to manifest in various ways; here, we focus on three imminent manifestations to illustrate its scope: Anticipatory Spillover occurs when participants expect LLMs to be present and adjust their behaviour accordingly. For instance, they might suspect that an interaction partner in a strategic game uses an LLM, or that the experimenter paired them with an LLM partner rather than a human one, and in response, change their behaviour in terms of cooperation, trust, or disclosure. Meanwhile, Residual Spillover can arise from prior exposure to LLMs in unrelated contexts. Such cross-domain effects can result from well-known mechanisms like linguistic or emotional priming and fixation, which have also been observed in other digital settings. For example, exposure to AI-suggested wording can shift people’s tone and language use beyond the moment of interaction^25^, and brief LLM exposure has been shown to reduce idea diversity and hinder later unassisted performance^26^. Similar carryover patterns have also been found in digital search and ideation tasks that narrow cognitive focus^27^. Within the context of Residual Spillover from LLMs, recent empirical work indicates that people’s spoken language has already shifted since the rollout of ChatGPT^18^, suggesting that we should expect both altered linguistic patterns in participant responses as well as signalling behaviours that seek to avoid overtly “LLM-like” phrasing. As yet another manifestation, Normative Spillover reflects broader cultural adjustments in response to the rising ubiquity of LLMs. Again, such adjustments may unfold in different directions: one plausible trajectory is that, as LLM-use becomes ever more normalised in everyday life, participants may increasingly perceive their use as acceptable, even in contexts where it is explicitly discouraged. Education offers an early illustration of this dynamic: here, the rapid uptake of LLM-powered tools by students and teachers has fuelled debates about academic integrity^28^, and about whether, and under what conditions, their use constitutes cheating^29,30^ or instead a beneficial, even equalising, assistance^31,32^. At the same time, these debates have heightened sensitivity to AI involvement and spurred efforts to articulate clearer norms around appropriate versus inappropriate uses^33^. In online behavioural research, such heightened sensitivity may ultimately feed back into the *LLM Pollution* cycle by shaping participants’ engagement in *Partial LLM Mediation* or *Full LLM Delegation*, while it could also motivate community efforts to define norms and safeguards against the threat.

This backdrop highlights that the three variants of *LLM Pollution* capture interrelated processes that are better understood in relation than isolation. Mitigation strategies should reflect their interdependence, as interventions aimed at one variant can inadvertently amplify another. For instance, tightening controls to curb *Full LLM Delegation* may heighten Anticipatory Spillover effects. Addressing *LLM Pollution* thus requires strategies that are effective, proportionate, and sensitive to participant experience. Ideally, such strategies will also be scalable, empirically testable, and resilient to the continued evolution of the threat. In the following, we share examples of measures we have found promising and which we hope can provide a basis for a continuous community effort to mitigate *LLM Pollution*.

### Mitigation strategies: a multi-layered response

Since efforts to detect *LLM Pollution* are already headed towards an arms race—as tools improve, so too do evasion strategies^24^—eliminating LLM-generated responses entirely is unlikely to be feasible. Instead, alongside recent commentary outlining detection strategies for AI-generated survey responses^34^, we propose pragmatic, multi-layered mitigation strategies aimed at raising the cost and lowering the feasibility of *LLM Pollution* across different levels of behaviour research ecosystems. This includes safeguards that individual researchers can implement in study design, greater responsibility on research platforms to ensure sample integrity, and broader efforts to foster community-wide norms that promote data quality. Crucially, different forms of *LLM Pollution* require tailored responses, as no single tool addresses all threats equally^6,35^.

Table 1 summarises a range of measures that individual researchers can adopt to mitigate or detect *LLM Pollution*. No single intervention is without concerns, and many rely on complex trade-offs between effectiveness and practical constraints. These measures vary in scope and function, but all aim to raise the cost and lower the feasibility of unwanted LLM involvement. For more details and examples of the measures, see Supplementary Information.

**Table 1:** List of possible measures for detecting and mitigating *LLM Pollution*

Measure	Example implementations	Responsibility	Purpose	Variants	Risks & concerns
Third-party bot protection solutions	Use Cloudflare Bot Management, reCAPTCHA, etc.	Platforms, Researchers	Prevention	LLM Delegation LLM Spillover	Locally-running or human-intervening agents can circumvent the protection.
Norm signalling to participants	Remind participants of expectations and ask for authenticity affirmation	Platforms, Researchers, Community	Prevention	LLM Mediation LLM Delegation LLM Spillover	Making LLM presence too salient can increase suspicion or reactivity among participants.
Multimodal presentation of instructions	Present illustrated images or tutorial videos	Researchers	Prevention	LLM Mediation LLM Delegation	Some participants and automation tools provide screenshots to vision-capable models.
Human-supervised or multimodal response collection	Block copying and pasting, require spoken or webcam-enabled responses, or conduct live sessions where participants provide verbal answers (e.g., via Zoom)	Researchers	Prevention	LLM Mediation LLM Delegation LLM Spillover	Audio and video recording might have privacy concerns despite its effectiveness.
LLM-specific comprehension checks	Present modified versions of Theory of Mind tests, visual illusion quizzes, etc.	Researchers	Prevention	LLM Mediation LLM Delegation	Comprehensive checks effective for some models are not necessarily applicable to others.
Honeypot questions	Add very small or white text with specific instructions for LLMs; implement prompt injection techniques	Researchers	Post-hoc detection	LLM Mediation LLM Delegation	Advanced LLM-based agents might be able to detect and ignore honeypot questions.
Behavioural logging and modelling	Use typing speed, contents, or mouse movements to identify polluted cases	Researchers	Post-hoc detection	LLM Mediation LLM Delegation	Automation tools might become capable of imitating human-like behaviours.
Commercial AI-generated text detectors	Apply GPTZero, undetectable AI, etc.	Researchers	Post-hoc detection	LLM Mediation LLM Delegation	Commercial models lack transparency and assurance against new models.
Institutional enforcement	Standardise policies and procedures for identifying, reporting, and refunding polluted cases	Platforms, Community	Prevention	LLM Mediation LLM Delegation LLM Spillover	Fairness between participants’ access and researchers’ control can be in tension.
Shared repository of best practices	Collect and disseminate reusable templates, code, etc.	Researchers, Community	Prevention, Post-hoc detection	LLM Mediation LLM Delegation LLM Spillover	Repositories may soon become outdated as model capabilities evolve.

One set of strategies focuses on preventative measures designed to increase friction for LLM use. Commercial bot protection tools like reCAPTCHA or Cloudflare are widely available and relatively easy to implement. These tools can block access from known LLM platforms or agents (e.g., OpenAI’s ChatGPT Agent), and the platforms themselves are less likely to override such protections for ethical reasons. However, locally run tools or hybrid user-agent models can often bypass these barriers by temporarily returning control to a human. Another line of defence is to vary the mode of instruction presentation by using screenshots, images, or short videos to reduce the ease of copying and pasting into LLMs. While this may limit casual use, vision-enabled models and screen-sharing tools can again circumvent such barriers. Relatedly, restricting input methods can make automation more difficult by means of disabling pasting, requiring typed responses, or blocking right-click functionality. Recent evaluations of explicit requests not to use LLMs and interface-based copy-paste restrictions indicate that these measures can reduce LLM-usage at least to some extent^6^. Also, using multimodal channels (e.g., collecting responses via voice input or recording video via webcam) may offer an additional layer of friction, as it is currently harder to automate reliably. In some cases, researchers may also opt to collect responses in real time through supervised sessions via Zoom, for example, where participants provide verbal answers that are directly recorded by experimenters. While this approach offers the highest level of verification, it substantially increases the human resources demands for labs. Importantly, all of these strategies must be weighed against usability and privacy concerns, particularly in longer studies or with vulnerable populations. Finally, behavioural research provides scientists with additional levers for LLM detection by leveraging task structures that probe cognitive processes rather than merely aggregate responses. For example, researchers may design prompts that contemporary LLMs struggle with, such as variations of visual illusions^36^ or of Theory of Mind questions^37^, combined with additional comprehension checks (see Supplementary Note S2.5 and Supplementary Figs. S2 and S3). While current models tend to overcorrect or produce obviously wrong answers in such tasks, these vulnerabilities are likely to diminish as models improve. Hence, again, such preventative strategies may offer only temporary protection unless regularly updated.

Beyond prevention, researchers can also take steps to identify LLM involvement after the fact. Honeypot questions, such as inserting white or inaccessible text, can detect whether a participant’s browser is scraping the entire page, as many LLM agents do. These traps exploit the tendency of automated tools to process all on-screen content indiscriminately, including elements that human users cannot see (see Supplementary Methods S2.6 for examples). They can be combined with specific prompt injection techniques, such as jailbreaking or prompt leaking^35^ (see Supplementary Methods S2.6 for examples and discussion). Recording behavioural data like typing speed, mouse movement, and tab switching can also flag suspicious patterns. While helpful, these signals can be mimicked by more advanced agents. Researchers may choose to run AI-generated text detectors on open-ended responses. These tools, such as GPTZero or Undetectable AI, can provide estimates of LLM involvement, but they often lack transparency and may quickly become outdated as models evolve.

Mitigating human reactivity effects that come to bear in *LLM Spillover* poses a distinct challenge, as interventions themselves can influence participant expectations. Simply informing participants that bot detection tools are in use may discourage casual or opportunistic reliance on LLMs, and explicit reminders that responses should reflect their own judgment can reduce mild or exploratory use. However, these interventions can also backfire by drawing attention to LLM presence and researcher scrutiny. In doing so, they may heighten participants’ self-consciousness or suspicions about other participants’ LLM usage, fuelling the very demand effects and signalling behaviours they aim to prevent. In this way, strategies designed to reduce *Partial LLM Mediation* and *Full LLM Delegation* may inadvertently exacerbate the *LLM Spillover* loop. To counteract this risk, mitigation must go beyond technical safeguards and reinforce shared norms around fairness, effort, and genuine participation. Establishing these norms may be crucial for re-anchoring expectations and sustaining the long-term integrity of online research.

As the burden on individual researchers to detect and prevent *LLM Pollution* continues to grow, platform- and community-level support will be essential to making mitigation scalable and sustainable^38^. Platforms hosting online research should take greater responsibility for ensuring data integrity. For example, by strengthening terms of service to prohibit unauthorised LLM use and by providing clearer participant guidance to align expectations. Features such as refund policies or abuse reporting tools may further incentivise responsible behaviour and motivate institutional investment in prevention. Beyond platform measures, fostering community-wide standards and practices is also important. Sharing knowledge, coordinating responses, and working toward common safeguards can reduce implementation burdens and improve consistency across the community. In the long-term, reinvesting in physical lab infrastructure or supervised environments may be necessary in cases where higher control is needed. Such environments offer a level of oversight over participants’ devices, attention, and behaviour that remains difficult to achieve through technical safeguards alone. However, given the costs and practical limitations of lab-based research, collaborations will again be key to ensuring feasibility and broader access.

Overall, no single strategy is sufficient on its own. Each comes with trade-offs, and their effectiveness depends on the study context, participant population, and evolving capabilities of LLMs. Still, when combined into a layered and adaptive approach spanning individual researchers, platform governance, and community-wide coordination, these strategies can meaningfully reduce the risk of *LLM Pollution* and help preserve the integrity of online behavioural research.

## Discussion

*LLM Pollution* presents an epistemic challenge, not just a technical one. It blurs the line between human and machine in the very data we use to understand human cognition and behaviour. Crucially, the threat is not always adversarial. In many cases, participants use LLMs to improve fluency, reduce effort, or navigate complex instructions—behaviours that may increasingly reflect the realities of LLM-usage in everyday life. In our framing, *LLM Pollution* is the cause, and its effects include systematic distortions that can compromise validity and interpretation. This growing entanglement complicates the question of what counts as pollution and demands more than just technical safeguards. Even when intentions are benign, LLM involvement can introduce systematic distortions that are difficult to detect, impossible to disentangle post-hoc, and as such often invisible to researchers.

We have identified three key variants by which LLMs can pollute online research. *Partial LLM Mediation* occurs when participants enlist LLMs to refine or generate responses. *Full LLM Delegation* describes agentic tools completing entire studies. *LLM Spillover* describes changes in participant behaviour due to second-order effects such as inflated expectations about LLM involvement in online research studies. Together, these variants undermine sample authenticity, distort treatment effects, and compromise internal and external validity.

Researchers must recognise that *LLM Pollution* is not merely an inconvenience, but a growing methodological challenge that demands careful and routine consideration in experimental design. Preserving data validity entails the integration of multi-layered detection and prevention systems, including measures from entry screening to output verification. Nevertheless, such safeguards must be adopted with special attention to the participant experience. While it is essential to filter out suspicious responses, this should not come at the cost of inadvertently alienating or excluding genuine respondents who are motivated to contribute meaningfully. If legitimate participants are repeatedly misclassified or confronted with confusing and opaque screening mechanisms, their trust in the research process may erode over time, thus diminishing their motivation to invest the effort needed to provide thoughtful data. Moreover, by making the presence of detection mechanisms salient, such interventions risk inflating participants’ beliefs about widespread LLM use, amplifying the very reactivity they aim to reduce.

While individual researchers can implement many safeguards, durable solutions require institutional and infrastructural support. Platforms that host human-subject research must play a more active role in protecting data integrity. Beyond platforms, coordination at the community level is equally vital. Sharing knowledge, aligning screening standards, and investing in shared infrastructure can reduce redundancy and lower barriers to implementation.

While our focus is on online behavioural experiments, *LLM Pollution* can also arise in other forms of data that researchers may use to address research questions, such as observational datasets based on online activity (e.g., social media posts), where parts of the data may already be generated or altered by LLMs. Addressing these broader cases is important, and future research should examine how *LLM Pollution* affects other types of data and research settings.

As LLMs become increasingly embedded in everyday life, their use in cognitive, communicative, and problem-solving tasks may no longer be an exception, but the norm. This raises a more fundamental question: at what point does LLM-assisted behaviour cease to be pollution and instead become part of the ecological baseline we must account for? While mitigation remains essential for preserving the integrity of current methods, the long-term challenge may lie in adapting our theoretical frameworks to a world where human reasoning is increasingly shaped by intelligent machines. In this context, understanding the second-order dynamics of an LLM-infused society^39–41^ will provide insights into the spillover effects in online research ecosystems.

Looking ahead, there are several priorities for further empirical work in this area. Beyond offering conceptual distinctions, our framework highlights the need for systematic tracking of the prevalence and development of *LLM Pollution* as agentic tools become increasingly accessible to participants. Moreover, there is a need for further evaluation of mitigation strategies and the creation of measurement tools for spillover effects. Future research can build on this Comment to test which safeguards are most effective, which situational and motivational conditions make *Partial LLM Mediation* most likely, how LLM involvement alters behaviour in practice, and how evolving participant norms reshape the boundaries between human and machine-generated data.

Safeguarding the foundations of online behavioural research in the age of LLMs requires ongoing attention, flexibility, and collective responsibility. If these efforts are taken seriously, *LLM Pollution* may become less of a threat and more of a challenge that behavioural science learns to manage, without losing its integrity.

## Supplementary information

Supplementary Information
